# Direct Nitrate Reductase Assay versus Microscopic Observation Drug Susceptibility Test for Rapid Detection of MDR-TB in Uganda

**DOI:** 10.1371/journal.pone.0019565

**Published:** 2011-05-09

**Authors:** Freddie Bwanga, Melle Haile, Moses L. Joloba, Emmanuel Ochom, Sven Hoffner

**Affiliations:** 1 Department of Medical Microbiology, School of Biomedical Sciences, Makerere University College of Health Sciences, Kampala, Uganda; 2 Department of Bacteriology, Swedish Institute for Communicable Disease Control, Solna, Sweden; 3 Department of Microbiology, Tumour and Cell Biology, Karolinska Institute, Stockholm, Sweden; Hopital Raymond Poincare - Universite Versailles St. Quentin, France

## Abstract

The most common method for detection of drug resistant (DR) TB in resource-limited settings (RLSs) is indirect susceptibility testing on Lowenstein-Jensen medium (LJ) which is very time consuming with results available only after 2–3 months. Effective therapy of DR TB is therefore markedly delayed and patients can transmit resistant strains. Rapid and accurate tests suitable for RLSs in the diagnosis of DR TB are thus highly needed. In this study we compared two direct techniques - Nitrate Reductase Assay (NRA) and Microscopic Observation Drug Susceptibility (MODS) for rapid detection of MDR-TB in a high burden RLS. The sensitivity, specificity, and proportion of interpretable results were studied. Smear positive sputum was collected from 245 consecutive re-treatment TB patients attending a TB clinic in Kampala, Uganda. Samples were processed at the national reference laboratory and tested for susceptibility to rifampicin and isoniazid with direct NRA, direct MODS and the indirect LJ proportion method as reference. A total of 229 specimens were confirmed as *M. tuberculosis*, of these interpretable results were obtained in 217 (95%) with either the NRA or MODS. Sensitivity, specificity and *kappa* agreement for MDR-TB diagnosis was 97%, 98% and 0.93 with the NRA; and 87%, 95% and 0.78 with the MODS, respectively. The median time to results was 10, 7 and 64 days with NRA, MODS and the reference technique, respectively. The cost of laboratory supplies per sample was low, around 5 USD, for the rapid tests. The direct NRA and MODS offered rapid detection of resistance almost eight weeks earlier than with the reference method. In the study settings, the direct NRA was highly sensitive and specific. We consider it to have a strong potential for timely detection of MDR-TB in RLS.

## Introduction

Tuberculosis (TB) continues to be a leading public health problem in the developing countries, with Sub Saharan Africa being hardest hit [1]. Besides HIV/AIDS, drug-resistance is now recognized as one of the major factors underlying the failure to control TB [2]. Drug resistance in *M. tuberculosis* (MTB) develops by sequential selection following exposure to TB drugs [3]. In most of the low income Sub-Saharan African countries, only first line drugs [isoniazid (INH) and rifampicin (RIF), Ethambutol (ETH) and Pyrazinamide (PZA)] are available for TB treatment. Thus, multi drug resistance (MDR) - defined as resistance to at least INH and RIF is currently the main concern. The prevalence of MDR-TB in Africa remains largely unknown but is estimated to be between 1–4% among new and 4–17% among re-treatment TB cases [4]. The high number of TB cases per year in each of the high burden African countries [1] by itself implies that even a limited prevalence of MDR-TB represents a significant pool of potentially infectious MDR-TB cases. Timely detection of these cases is crucial for patient management and control of further MDR transmission [5].

Indirect susceptibility testing on Lowenstein-Jensen (LJ) medium is the most common method for detection of TB drug resistance in Africa. With this method, results take 2–3 months and during this period patients are given inappropriate drug regimens with poor responses and they continue to spread MDR strains, which might be causing MDR-TB outbreaks [6]. Commercial liquid culture techniques, such as the Mycobacterium Growth Indicator Tube (MGIT 960: Becton Dickinson, Sparks, Maryland) and line probe assays [7]–[8] allow more rapid detection of resistance, and have been recommended by the WHO [9]–[10]. However, the investment and recurrent costs is an obstacle for the broad implementation of these techniques in the resource-limited settings (RLSs) of Africa. Therefore, the need for a rapid, affordable, accurate and easy to use test for MDR-TB in RLSs remains a priority.

The Nitrate Reductase Assay (NRA) and the Microscopic Observation Drug Susceptibility (MODS) are two of the most promising rapid tests for MDR-TB proposed for RLSs. Both techniques have been reported to be low cost in-house assays that can be applied directly on smear positive sputum [11]. Resistance detection with the NRA is based on visual observation of a pink to purple color in a culture tube upon addition of the so called Griess reagent, due to nitro-reductase enzymes in metabolically active mycobacterial cells converting nitrate to nitrite [12]. MODS relies on microscopic observation of characteristic cord-like structures in the drug-containing wells of a tissue culture plate where resistant MTB cells are growing [13].

In 2009, we conducted a meta-analysis of studies of the direct NRA and MODS, and the pooled data showed high sensitivity and specificity for detection of resistance to RIF and INH [11]. The direct NRA has been studied in Brazil, India and Nigeria with good results [14]–[16], but these studies had limitations. For example in the Brazil study, the direct proportion method was the reference test, while in Nigeria only 20 sputum samples were studied. The World Health Organization (WHO) in July 2010 recommended the use of NRA and MODS to screen for MDR-TB in RLSs, but the available data to support the direct NRA was admittedly limited [17]. It is of priority to obtain sufficient data on these tests before full scale recommendation of their implementation in Africa.

In this study we provide more recent data and field experience with the NRA and MODS assays in the East African country of Uganda, a typical RLS. The assays were prospectively compared side by side for interpretable susceptibility results, contamination rates, sensitivity and specificity, time to results and cost per sample on a consecutive population of previously treated TB patients attending a TB clinic in Kampala. The study was approved by the Research and Ethics Committee of Makerere University College of Health Sciences Kampala, Uganda.

## Methods

### Study settings

The study was conducted at Mulago National Referral Hospital and at the National Reference Laboratory (NRL), Kampala Uganda. Mulago is a 1500-bed tertiary hospital belonging to the ministry of health, Uganda. With its free medical care, the hospital is particularly attractive for the peri-urban low income population around the capital Kampala where the TB incidence is highest. The hospital has a TB treatment centre where most TB suspects and microscopy-confirmed patients are referred for care. Around 4 500 patients are treated at the centre annually, 15–20% of whom are estimated to be re-treatment cases (Mulago Hospital TB register, 2006). About one kilometer away from Mulago is the NRL, which is a P2 TB laboratory facility belonging to the National TB Control Program (NTP). At the beginning of this study, the LJPM was the only assay for DST used at the NRL. The laboratory successfully participates in external quality assurance under the WHO supranational reference (SNRL) network.

### Study patients

Previously treated (re-treatment) TB suspects - return-after-default, treatment failures and relapses [3] were studied. Only those who were positive at Ziehl-Neelsen (ZN) smear microscopy were recruited into the study. A sample size of 250 smear positive patients was calculated using a simple nomogram - a statistical tool for calculation of sample size in diagnostic studies [18]. This calculation was based on a minimum required sensitivity of 95% for a direct MDR-TB test, 95% confidence interval of +/−7 and based on an estimated prevalence of MDR-TB of 15% among the re-treatment TB cases at Mulago hospital.

### Patient screening and recruitment

**Figure 1:**
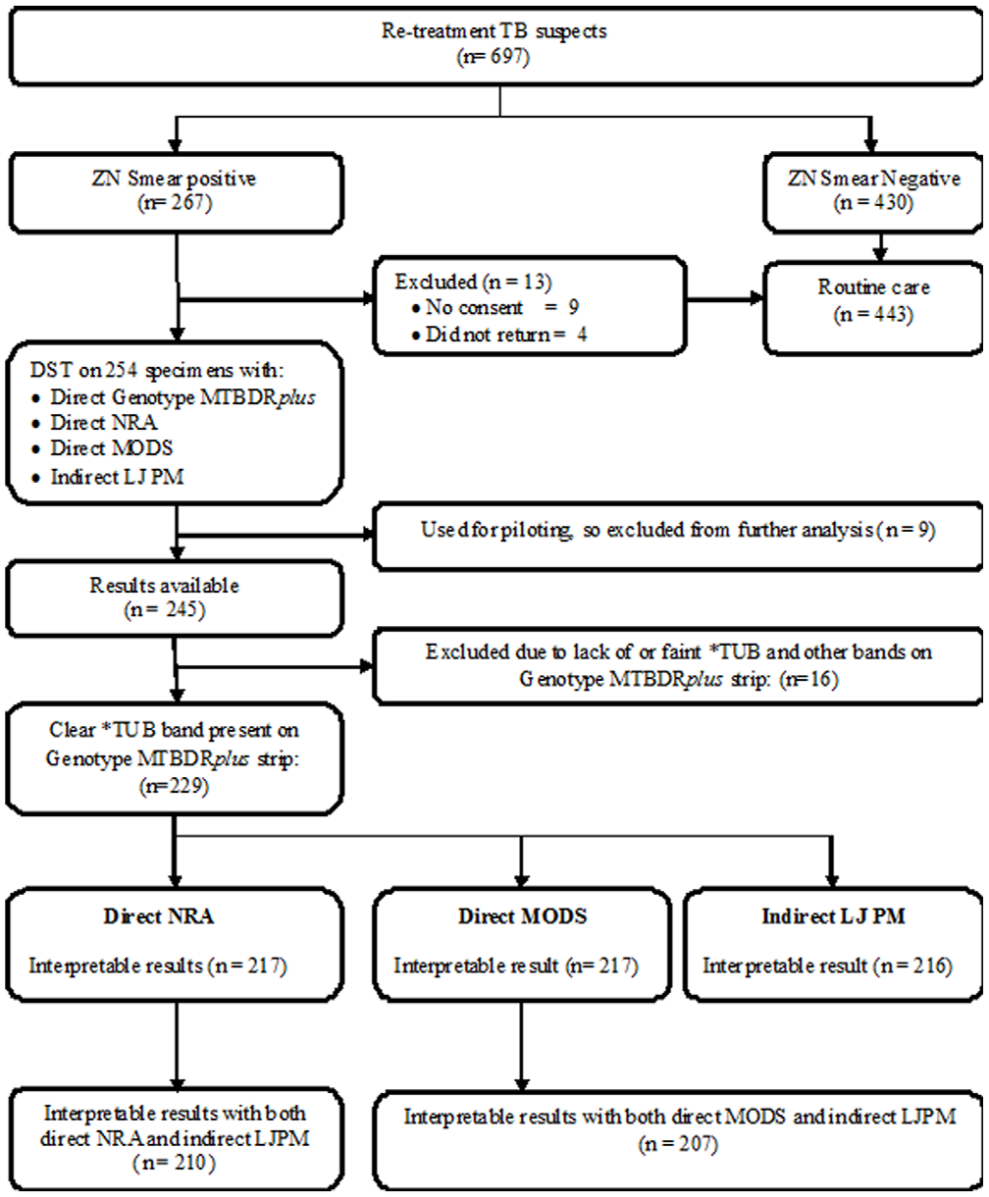
Patient screening, recruitment and laboratory assays.

Over an 18-months period beginning February 2008, routine ZN smear microscopy was done on at least two sputum specimens from all 697 re-treatment TB suspects reporting at the TB clinic (see figure 1). Of these, 267 (38%) were positive for acid fast bacilli, and they were requested to consent to the study irrespective of the smear grade [19]. Of these, 254 (95%) gave written consent to join the study. Two or three spot sputum specimens were then collected from each of these patients in 50 ml polypropylene tubes, before initiation of the WHO standard category II drug regimen [20]. Samples were packaged according to packing instruction 650 for Category B specimens [21] and transported at room temperature to the NRL. In case of delays of more than 2 hours, samples were kept at the clinic at 4–8°C until transported.

### Specimen processing and inoculum preparation

Specimens were processed immediately at the NRL, but a few specimens were kept at 4–8°C within the NRL for one or two days if brought in late on Friday evening. Each of the specimens was processed individually with the *N*-acetyl-*L*-cysteine–NAOH–sodium citrate method with NAOH at final concentration of 1.5% instead of the conventional 4% [22]. It is now routine practice at the NRL to process sputum with 1.5% NAOH final concentration to minimize the rampant culture contamination. The sediment in each tube was reconstituted with phosphate buffered saline (PBS) to 2.5 ml, mixed well and then pooled into one tube that served as common inoculum source for all subsequent tests.

### Direct nitrate reductase assay (NRA)

The LJ-NRA medium was prepared in-house. Mineral salts, homogenized egg solutions and malachite green were mixed as in the preparation of LJ medium. Potassium nitrate was added at a concentration of 1000 µg/ml. INH and RIF (Sigma®) stocks were prepared as previously described [23], and were included in the medium at 0.2 µg/ml and 40 µg/ml, respectively. To keep the test less laborious, before inoculations, no further sample dilutions were made. Instead, three-hundred microlitres of the sediment was inoculated on each of three drug-free controls (day 10, 14 and 21), and on the INH and RIF- tubes and incubated at 37°C. On the 10^th^ day, 200 µl of Griess reagent (a solution of hydrochloric acid 50% (vol/vol), sulfanilamide 0.2% wt/vol, and N-(1-naphtyl)ethylene-diamine dihydrochloride 0.1% (wt/vol) mixed in ratio of 1∶ 2∶ 2) was added to one control tube in class II bio safety cabinet (BSC) in a bio safety level 2 facility. If a pink to purple color developed, the reagent was also added to the two drug containing tubes. A pink-purple color in the drug tube indicated resistance. If none or only a faint pink color developed in the control tube, the slopes were re-incubated until the 14^th^ or 21^st^ day when Griess reagent was added to the second or third control tube, respectively, and then to the drug tubes.

### MODS assay

This assay was performed in a 24-well plate. Each well contained 700 µl of Middlebrooke 7H9 broth, 100 µl of a cocktail of polymyxin B, Amphotericin B, Nalidixic acid, trimethoprim and azlocillin (PANTA: BD®), 100 µL of solutions of INH 1 µg/ml or RIF 10 µg/ml and 100 µl of the processed specimen, giving a final volume of 1 ml/well, and critical concentrations of 0.1 µg/ml INH and 1 µg/ml RIF. A sterility control well with 7H9 broth-PANTA, and a growth control well with these plus the inoculum was included for each sample. Plates were sealed with tape and ziplock bags and incubated at 37°C. Plates were examined under an inverted microscope at ×20 and ×40 for cord-like structures on days 7, 10, 14 and 21. Daily readings were not practical in the study settings with few laboratory staff. For interpretability of results, the positive control well had to show cordlike structures while the sterility well showed no cords. A strain was considered resistant if cord-like structures were observed both in drug-free and drug-containing wells, and susceptible if cords were seen only in the drug-free controls.

### Indirect LJPM

The reference test was performed and interpreted according to standard procedures with the recommended critical concentrations of 0.2 µg/ml INH and 40 µg/ml RIF [24].

### Speciation and testing for discrepant results

All samples in this report were also tested with the Genotype® MTBDR*plus* assay (Hain Lifescience GmbH, Germany) to confirm the presence of MTB complex band [7]. Results of the Genotype® MTBDR*plus* test were also used to cross-check discordant results. This test detects mutations in the 81-bp hot spot region of the *rpoβ* gene for RIF resistance and in the *katG* gene or *inhA* promoter region for INH resistance [7].

### Repeat testing

A portion of the inoculum was frozen at minus 20°C, to be used if initial direct DST with the NRA, MODS or Genotype® MTBDR*plus* assay were un-interpretable.

### Time to results (TTR)

The dates of DST inoculation and reading of interpretable results for each sample were recorded and the days to results were calculated for the NRA, MODS and LJPM assays. Interpretable results referred to either ‘resistant’ or ‘susceptible’. Un-interpretable results referred to results such as ‘no growth’ or ‘contaminated tube/well’ where no result could be obtained even after repeat testing.

### Cost estimation of the direct NRA, MODS and LJPM

An estimation of the costs of laboratory supplies and consumables were performed based on prices given by a local supplier and Fisher Scientific® UK catalog 2009–2010. We added an estimated 15% surcharge to cover shipping costs. Salary and other indirect costs were not assessed.

### Data analysis

Nine samples were used for piloting the processes/methods, thus final data analysis was done on 245 specimens (see figure 1). Frequency as well as 2 by 2 tables and *kappa* agreements were generated in SPSS 11.0 for windows. Sensitivity, specificity and confidence intervals were analyzed with the meta-disc software.

## Results

Detailed DST results of the LJPM, NRA, MODS and Genotype MTBDR*plus* are shown in Table S1.

### Interpretable susceptibility results

Using the Genotype® MTBDR*plus* assay (Hain Lifescience GmbH, Germany), 229 (93%) of the 245 studied specimens showed a clear MTB band on the strip, confirming them as members of the MTB complex. With the direct NRA, 217 (95%) of the 229 results were interpretable - 86% at initial testing. Repeat NRA testing was due to contamination, indeterminate results or lack of growth in 18(8%), 9(4%) and 4(2%) samples, respectively. With the direct MODS assay, 217 (95%) of the 229 results were interpretable - 91% at initial testing. Repeat MODS testing was due to lack of growth in the growth control well 11(5%), contamination 7(3%), and drying in wells 2(1%). Lack of sufficient growth and contamination accounted for the totally un-interpretable results (5% of all samples) with both tests (see Table 1).

**Table 1:** Interpretable and Un-interpretable susceptibility results, (n = 229).

Results	Direct NRANo. (%)	Direct MODSNo. (%)	Indirect LJPMNo. (%)
***Interpretable results:***			
Susceptible to both RIF & INH	149 (65)	143(62)	151 (66)
MDR	39 (17)	44(19)	39 (17)
INH Mono-resistant	24(11)	24(11)	22 (9)
RIF Mono-resistant	5 (2)	6(3)	4 (2)
**Subtotal**	**217 (95)**	**217 (95)**	**216(94)**
***Un-interpretable results:***			
Insufficient growth	8 (3)	10(4)	6 (3)
Contaminated culture or DST tube/well	4 (2)	2(1)	7 (3)
**Subtotal**	**12 (5)**	**12(5)**	**13 (6)**
**Total**	**229 (100)**	**229 (100)**	**229 (100)**

INH = Isoniazid; LJ PM = Lowenstein-Jensen proportion method; MDR = Multidrug resistant; MODS = Microscopic Observation Drug Susceptibility; NRA = Nitrate Reductase Assay; RIF = Rifampicin.

### Sensitivity and specificity of the direct susceptibility testing

Of the 217 specimens with interpretable direct NRA or MODS results, 210 and 207 were interpretable with the LJPM, respectively, and were used in the analysis for sensitivity and specificity. Sensitivity was defined as the proportion of drug resistant strains correctly identified by the study tests (true positive), and specificity as the proportion of susceptible strains correctly identified (true negative).

### Direct NRA

Sensitivity, specificity and *kappa* agreement for detection of MDR were, 97%, 98% and 0.93, respectively. The Genotype® MTBDR*plus* agreed with the NRA for the lone sample regarded as non-MDR with the NRA but as MDR with the LJPM. If this sample was regarded as truly non-MDR, the sensitivity of NRA would potentially increase to 100%. For the three specimens classified as MDR with the NRA but non-MDR with the LJPM, the Genotype® MTBDR*plus* agreed with the LJPM, but two of these three specimens were mono-resistant to isoniazid with all three tests.

### Direct MODS

Sensitivity, specificity and *kappa* agreement for MDR-TB detection was 87%, 95% and 0.78, respectively. Of the five specimens categorized as non-MDR with the MODS but MDR with the LJPM, the Genotype® MTBDR*plus* test agreed with the MODS in only two cases. If these two specimens were to be included among the true MDR strains, sensitivity of MODS would potentially increase to 92%. Of the nine specimens categorized as MDR with MODS but non-MDR with LJPM, the Genotype® MTBDR*plus* test agreed with MODS in only one case; eight specimens remained non-MDR by the Genotype® MTBDR*plus* test and they were all susceptible to rifampicin in agreement with the LJPM.

### Time to results

Time to results was computed for specimens with interpretable DST results of both the study test and the Genotype® MTBDR*plus i.e.* 217 specimens for either NRA or MODS. The median time was 7 days (range 5–38 days) for MODS, 10 days for NRA (range 10–23 days) and 64 days (range 39–215 days) for LJPM. With MODS, 62% of the results were available by day 7 but by the 14th day, both MODS and NRA assays had 92% of the results available (see Table 2).

**Table 2:** DST results obtained within specified days.

Results within	MODSNo. (Cumulative %)	NRANo. (Cumulative %)
7 days	135 (62)	-
10 days	45 (83)	160 (74)
14 days	19 (92)	40 (92)
After 14days	18 (100)	17 (100)
**Total**	**217 (100)**	**217 (100)**

MODS = Microscopic Observation Drug Susceptibility; NRA = Nitrate Reductase Assay; RIF = Rifampicin.

### Cost estimates

The estimated cost of direct susceptibility testing with the NRA and MODS was $3.58 and $5.56, respectively (see Table 3).

**Table 3:** Cost estimation of tests.

Laboratory activity	Cost, USD		
	Direct NRA	Direct MODS	Indirect LJPM
Sputum processing	2.15	2.15	2.15
Culture before DST	NA	NA	0.47
Inoculation of Direct DST	0.53	2.69	NA
Inoculation of indirect DST	NA	NA	0.96
Reading Direct DST	0.43	NA	NA
**Subtotal**	**3.11**	**4.84**	**3.58**
Shipping etc.(15% of direct costs)	0.47	0.73	0.54
**Total cost**	**3.58**	**5.56**	**4.12**

*DST = Drug susceptibility testing*; LJ PM = proportion method on Lowenstein-Jensen Medium; MODS = Microscopic Observation Drug Susceptibility; NA = *Not Applicable*; NRA = Nitrate Reductase Assay; *USD = United States dollar*.

## Discussion

The number of TB cases arising annually in Sub Saharan Africa is alarming (>300 cases per 100, 000 population per year) [1]. The National TB control programs are however unable to routinely screen or do surveillance for MDR-TB due to lack of affordable rapid tests. The overall aim of this study was to compare two low cost direct DST assays, the NRA and MODS. We analyzed the proportion of interpretable results obtained at initial testing, sensitivity, specificity, time to results, contamination rates, and cost per sample. Interpretable results were seen in over 90% of the samples with either assays, most of them at initial testing. Moreover, results in this study show higher proportion of interpretable results than the previous reported 80–83% of samples with direct NRA [25]–[27] and 89% with MODS [13]. One reason for this could be that we repeated the tests for all initially un-interpretable results, unlike previous authors who did not. However, even in our study, interpretable results obtained at initial testing with NRA, MODS and LJPM were 186/217 (86%), 197/217 (91%) and 189/216 (88%) for LJPM, respectively. These findings suggest that these assays can be easy to perform in RLS. The rapid detection of drug resistant TB with the direct assays would allow a timely decision on therapy. For the few samples, without interpretable results at initial testing, the main reason was contamination for direct NRA and lack of growth for MODS. In the MODS assay, PANTA is included in the medium, which is not the case for NRA, explaining the difference in contamination rates. Contrary to the much feared problem of contamination with direct DST, insufficient growth, not contamination was the main reason for total failure to obtain results (Table 1).

### Direct Nitrate Reductase Assay

Sensitivity, specificity and *kappa* agreement for detection of resistance to RIF, INH and their combination (MDR-TB) was excellent (Table 4). These findings are in agreement with earlier reports [11], [28] implying that the direct NRA for rapid detection of MDR-TB can be consistently good across several study settings. Moreover, for the lone specimen classified as non-MDR with the NRA but MDR with the LJPM, the Genotype® MTBDR*plus* test agreed with the NRA results, potentially increasing the sensitivity of direct NRA to 100%. For the three specimens classified as non-MDR with the LJPM but MDR with the NRA, the Genotype® MTBDR*plus* agreed with the LJPM, but two of these three specimens were resistant to isoniazid with all three tests. The excellent sensitivity, specificity, and ease of implementation show direct NRA to be technically suitable for rapid diagnosis of MDR-TB in low income high TB burden countries. Since most of the retreatment patients have non-MDR disease, this highly sensitive test should be used to rapidly detect the MDR cases and to confidently exclude the majority without MDR disease. Early management of detected MDR cases would begin as further testing continues on only the MDR cases to confirm their status, thus optimizing the use of scarce resources.

**Table 4:** Technical performance of the direct NRA (n = 210).

NRA		LJPM		Sensitivity%	Specificity%	*Kappa* Agreement	PPV%	NPV%
		R	S					
RIF	R	40	4	98 (95% CI 87–100)	98 (95% CI 94–99)	0.93	91	99
	S	1	165					
INH	R	56	6	93 (95% CI 83–98)	96 (95% CI 91–99)	0.88	90	97
	S	4	144					
RIF+INH(MDR)	R	36	3	97 (95% CI 85–100)	98 (95% CI 95–100)	0.93	92	99
	S	1	170					

INH = Isoniazid; LJPM = Lowenstein-Jensen proportion method; MDR = multidrug resistant; NPV = Negative predictive value; NRA = Nitrate Reductase Assay; PPV = positive predictive value; R = resistant; RIF = Rifampicin; S = susceptible.

### MODS assay

This test gave good sensitivity and specificity for detection of resistance to RIF, INH and MDR-TB but the overall performance was somewhat lower than for NRA, with *kappa* agreement for MDR-TB of 0.78 (Table 5). These MODS results are somewhat less good compared to earlier reports, where sensitivity and specificity ranged from 92%–100% [11]. Additionally, more cases of false MDR-TB were detected with the MODS assay compared to the NRA. In our experience MODS false resistant results could happen if artifacts are interpreted as cords since the only identification test used was visual “cord formation”. It appears that failure to distinguish artifacts from cords and non-TB Mycobacterial growth from MTB cords can lead to a false resistant interpretation. Earlier reports also found false positive results with the MODS assay [29]. Recent modification of MODS assay such as addition of a well with a Para-Nitrobenzoic Acid (PNB) – a reagent that prevents growth of MTB complex but not other mycobacteria would help to minimize false resistant results [30]. The MODS assay is however, potentially an economical test in laboratories with many samples but less incubator space since one plate is adequate for at least 4 samples. However, its lower technical performance compared with NRA in the study setting is a disadvantage.

**Table 5:** Technical performance of the direct MODS assay (n = 207).

MODS		LJPM		Sensitivity%	Specificity%	*Kappa* Agreement	PPV%	NPV%
		R	S					
RIF	R	36	11	88 (95% CI 73–96)	93 (95% CI 88–97)	0.77	75	97
	S	5	155					
INH	R	53	11	90 (95% CI 79–96)	93 (95% CI 87–96)	0.80	83	96
	S	6	137					
RIF+INH(MDR)	R	32	9	87 (95% CI 71–96)	95 (95% CI 90–98)	0.78	78	97
	S	5	161					

S = susceptible; R = resistant; LJPM = proportion method on Lowenstein-Jensen Medium; MDR = multidrug resistant; NPV = negative predictive value; PPV = positive predictive value; MODS = Microscopic Observation Drug Susceptibility; RIF = rifampicin; INH = isoniazid.

### Time to results

As expected, both direct tests were far more rapid than indirect LJPM but with MODS having the shorter median time to results, *i.e.* 7 days, but 10 with NRA. Additionally, the proportion of results obtained within 10 days was slightly higher for MODS (83%) than for NRA (74%). However, by day 14 both tests had an equal proportion of interpretable results (92%).

Previous direct NRA studies reported fewer proportions of results within 10 days compared to our study findings [14]–[16], [25]–[27], [29]. In those studies, the control tubes received a 1∶10 diluted inoculum while in our study, the same undiluted inoculum was used in both the controls and drug tubes. Differences among studies could also be due to different positivity level of AFB in the sputums since patients in RLS tend to report with advanced disease. Nevertheless, majority of earlier studies also reported time to results varying from 10–15 days for around 80% of the samples. Given the high sensitivity and specificity of direct NRA, a median time of 10 days appears reasonable for an accurate MDR diagnosis in a RLS. Moreover, 92% of interpretable results were obtained within 14 days with NRA as it was for MODS (see Table 5).

According to the WHO, validated methods that detect resistance within 2–3 weeks can be recommended for rapid testing when molecular methods are not available [20]. Thus, our results comply with the WHO's recommendation of rapid DST of *M. tuberculosis* in settings where molecular tests are unavailable. The NRA, which was very accurate in the study setting would represent a significant improvement in MDR diagnosis from the public health and individual patient perspectives compared with the indirect LJPM, which in our study had a median TTR of 64 days.

### Cost per sample

The direct consumables cost for sputum processing, inoculation and reading of the susceptibility test were estimated. All three tests required the same sputum processing cost of $2.15. The NRA uses almost the same consumables as the LJPM except for the addition of potassium nitrate in the medium and later addition of the Griess reagent. However, both direct tests exclude the need for prior culture to isolate *M. tuberculosis* before performing DST, which explains the lower cost of direct NRA compared to the indirect LJPM (estimated costs $4.12 per sample). In our setting the direct NRA was cheapest ($3.58 per sample). The MODS assay requires culture plates, growth supplements and PANTA that may inevitably increase the cost per test ($5.56). The MODS assay also requires the use of an inverted microscope, which is not available in most TB laboratories in RLS. More recently, a less costly inverted microscope has been designed and in the future the MODS assay might cut the investments costs [31]. The differences in reported costs in our and earlier studies [13] clearly illustrate the difficulty involved in cost comparison in different settings.

### Bio safety

A validated and well maintained class II BSC is needed for the NRA test. The use of a BSC minimizes significantly the risk of aerosol inhalation of harmful aerosols.

For the MODS assay since the test is based on liquid media it is even more important to perform all the procedures from sample preparation, plate inoculation and plate sealing in a class II BSC. Extra care should be taken during plate sealing to avoid spillage and cross contamination between wells. In our experience, parafilm cracked during incubation and should not be used.

WHO recommends that direct DST, with NRA or MODS can be carried out in a laboratory with restricted access and a class II BSC as minimum requirements which is supported by others [17], [32]. Most TB laboratories in RLSs are very basic often the only bio safety equipment is a class II BSC. In a well managed laboratory, with appropriate bio safety routines an acceptable bio safety level can be achieved and the direct DST can be implemented.

### Conclusion

The direct NRA and MODS gave interpretable DST results in over 90% of smear positive sputum samples mostly within 14 days. In the study settings, the direct NRA was highly sensitive, specific and somewhat cheaper. We consider the direct NRA to have a strong potential for the direct detection of MDR-TB in resource-limited settings.

## Supporting Information

Table S1 Susceptibilty results of the LJPM, NRA, MODS and Genotype® MTBDR*plus*.
(XLSX)
